# Late Complication of Quadricuspid Aortic Valve: Early Moderate to Severe Aortic Regurgitation

**DOI:** 10.7759/cureus.27312

**Published:** 2022-07-26

**Authors:** Adeyinka Adeniyi, Sandra Abadir, Paul Douglass, Chantelle Brown

**Affiliations:** 1 Internal Medicine, Wellstar Atlanta Medical Center, Atlanta, USA; 2 Cardiology, Wellstar Atlanta Medical Center, Atlanta, USA

**Keywords:** heart valve disease, aortic valve, aortic valve replacement, aortic regurgitation, quadricuspid aortic valve

## Abstract

Quadricuspid aortic valve (QAV) is a rare congenital cardiac anomaly. A normal aortic valve has three cusps, but cases of unicuspid, bicuspid, and quadricuspid aortic valves have been reported. Although QAV usually appears as an isolated congenital anomaly, it may also be associated with other heart conditions. In comparison to the bicuspid aortic valve (BAV) that results in aortic stenosis by the early 50s due to age-related early calcification, this case series suggests that patients with QAV are likely to develop moderate to severe aortic regurgitation in their late 40s or early 50s. Most patients with QAV require tricuspidalization, which is the preferred method for QAV surgical repair, especially in patients with associated aortic regurgitation. The condition was previously diagnosed intraoperatively or postpartum. Today, with imaging modalities like transthoracic echocardiography (TTE), transesophageal echocardiography (TEE), and cardiac magnetic resonance imaging, more cases of QAV have been diagnosed in asymptomatic individuals. We present a case series of a previously healthy 49-year-old male and a 47-year-old female who had similar presentations of acute congestive heart failure (CHF). An echocardiogram confirmed that both patients had heart failure with reduced ejection fraction, dilated cardiomyopathy, QAV, and moderate to severe aortic valve regurgitation on echocardiogram. The male patient had an ejection fraction (EF) of 30-35% and a QAV with partial fusion of the leaflets, resulting in a functionally bicuspid aortic valve, while the female patient had an EF of 25-30% with what appears to be a type III QAV according to Nakamura et al. classification. The purpose of this case series is to highlight another potential late complication of congenital QAV.

## Introduction

A normal aortic valve has three cusps, but cases of unicuspid, bicuspid, and quadricuspid valves have been reported. The etiology of these anomalies is unknown, but different theories currently exist. Quadricuspid aortic valve (QAV) is a congenital cardiac anomaly found in less than 0.01% of autopsies [1]. Recently, there has been increased awareness of QAV because of the availability of imaging modalities like a transthoracic echocardiogram (TTE) and transesophageal echocardiogram (TEE) [2]. Early diagnosis and regular follow-up are needed because most patients will eventually require surgical intervention to fix the aortic insufficiency [2].

## Case presentation

Case 1

A 49-year-old male with a past medical history (PMHx) of uncontrolled insulin-dependent diabetes mellitus (IDDM) with a hemoglobin A1C of 14% presented with one week of progressive edema involving bilateral lower extremities, thighs, and scrotum. Associated symptoms included abdominal distention, a three-day history of cough productive of whitish phlegm, 18-pound weight gain, orthopnea, and dyspnea on exertion after walking a block. The patient denied chest pain, palpitations, syncope, nausea, vomiting, fever, or chills. He never smoked tobacco, used illicit drugs, or drank alcohol.

His vital signs on admission were within normal limits except for a blood pressure of 144/71 mm Hg. Physical examination was significant for bilateral inspiratory crackles on lung auscultation. Cardiovascular examination revealed a normal rate with regular rhythm and a diastolic murmur in the left lower sternal border that increased with expiration and leaning forward, jugular venous distention, and 2+ bilateral pitting edema in the lower extremities extending up to the thighs. An abdominal examination was positive for mild distention without guarding and rebound. A genitourinary examination showed non-tender bilateral scrotal edema. A complete blood count (CBC) and basic metabolic panel (BMP) were within normal limits. Troponin was elevated at 0.107 ng/ml (was not trended as the patient denied chest pain; elevation was suspected to be due to strain related to heart failure). Brain natriuretic peptide (BNP) was elevated at 1393 pg/ml.

**Figure 1:**
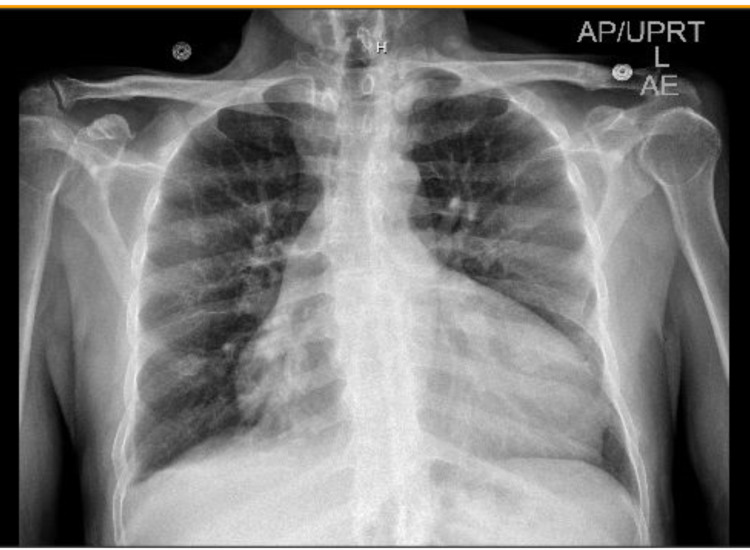
Standard posteroanterior chest X-ray CXR of a 49-year-old male who presented with a week of edema involving bilateral lower extremity, thighs, and scrotum, abdominal distention, cough productive of whitish phlegm, 18 pounds weight gain, orthopnea and dyspnea on exertion. The CXR shows enlarged cardiac silhouette, cardiomegaly, and pericardial effusion.

**Figure 2:**
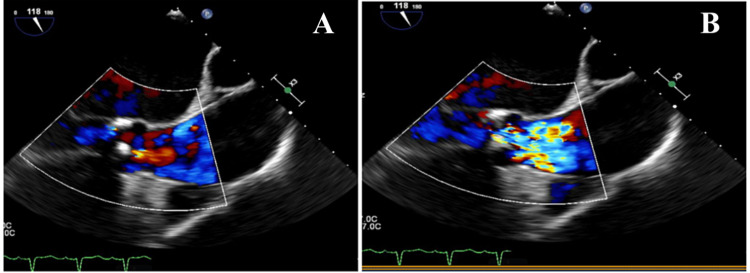
Transthoracic echocardiogram showing aortic regurgitation during diastolic phase (A) and systolic phase (B)

**Figure 3:**
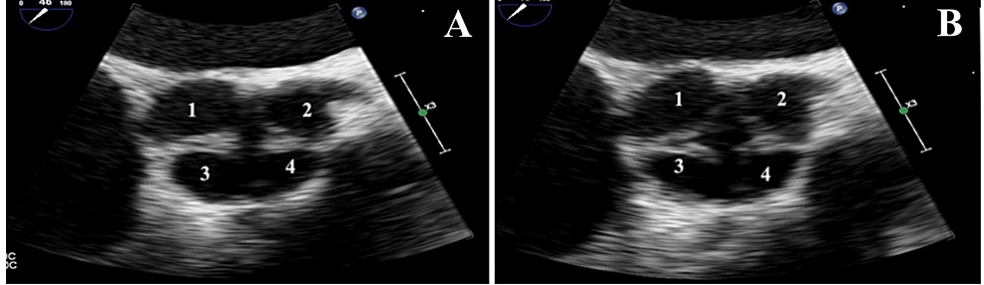
Transesophageal echocardiogram taken four days after admission showing quadricuspid valve (1-4) during isovolumetric contraction phase (A) and early systolic phase (B)

An electrocardiogram (ECG) revealed sinus rhythm with premature atrial complexes, possible left atrial enlargement, left axis deviation, and poor R wave progression. A chest X-ray (CXR) (Figure 1) showed an enlarged cardiac silhouette, cardiomegaly, and pericardial effusion. A cardiac stress test revealed fixed defects in the basal and mid-inferior regions without reversible ischemia. A TTE showed an ejection fraction (EF) of 30-35% with moderate to severe aortic regurgitation (Figure 2). Four days after his admission, a TEE (Figure 3) was done and was significant for EF of 20-25% with severe diffuse hypokinesis. The aortic valve showed quadricuspid with partial fusion of leaflets, resulting in a functionally bicuspid valve. The aorta was mildly dilated, measuring 4.1 cm at the level of the mid-ascending aorta. There was malcoaptation of the leaflets with a resultant central gap. Additionally, there was severe aortic regurgitation directed centrally in the left ventricular outflow tract with a vena contracta of 0.78 cm. A cardiac catheterization performed during the hospital stay showed mild non-obstructive coronary artery disease with moderate-severe pulmonary hypertension.

The patient was managed with guideline-directed medical therapy (GDMT) and placed on fluid restriction, low sodium diet, daily weight, and input/output monitoring. After optimal management of the patient's heart failure and IDDM, the cardiothoracic surgeon was consulted. The patient had bioprosthetic aortic valve replacement surgery as an outpatient on day 19 post-discharge from the hospital due to severe aortic insufficiency with congestive heart failure (CHF) and severe left ventricular dysfunction with an EF of 20-25%.

Case 2

A 47-year-old African American female with no significant PMHx presented to the emergency department with a one-week history of worsening shortness of breath with an associated cough productive of white blood-tinged sputum. She reported diaphoresis, nausea, diarrhea, orthopnea, palpitations, chest pain, and dyspnea on exertion. She denied fever, chills, syncope, emesis, recent travel, or recent sick contact. She denies ever smoking, drinking, or using illicit drugs.

Vitals on admission revealed a blood pressure of 182/87 mm Hg, a respiratory rate of 24 breaths per minute, and a heart rate of 109 beats per minute. Cardiovascular examination revealed a normal rate with regular rhythm with a grade 3/6 diastolic murmur heard in the left lower sternal border; the point of maximum impulse was displaced laterally with the S3 gallop audible in the mitral region. CBC and BMP were within normal limits. Troponin was 0.08 ng/ml (increasing to 0.17 ng/ml then to 0.19 ng/ml) and BNP was 4902 pg/ml.

**Figure 4:**
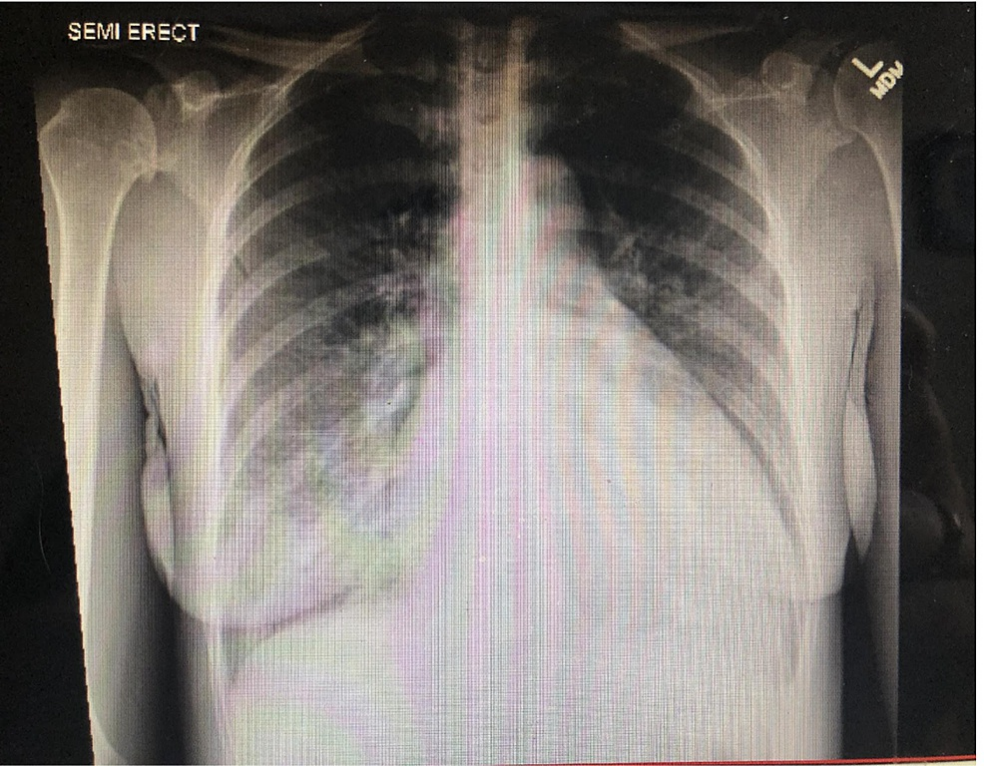
Standard posteroanterior chest X-ray CXR of a 47-year-old female who presented with one week of shortness of breath, cough productive of white blood tinged sputum, nausea, diarrhea, diaphoresis, orthopnea, palpitations, chest pain, and dyspnea on exertion. The CXR showed cardiomegaly and pulmonary edema.

**Figure 5:**
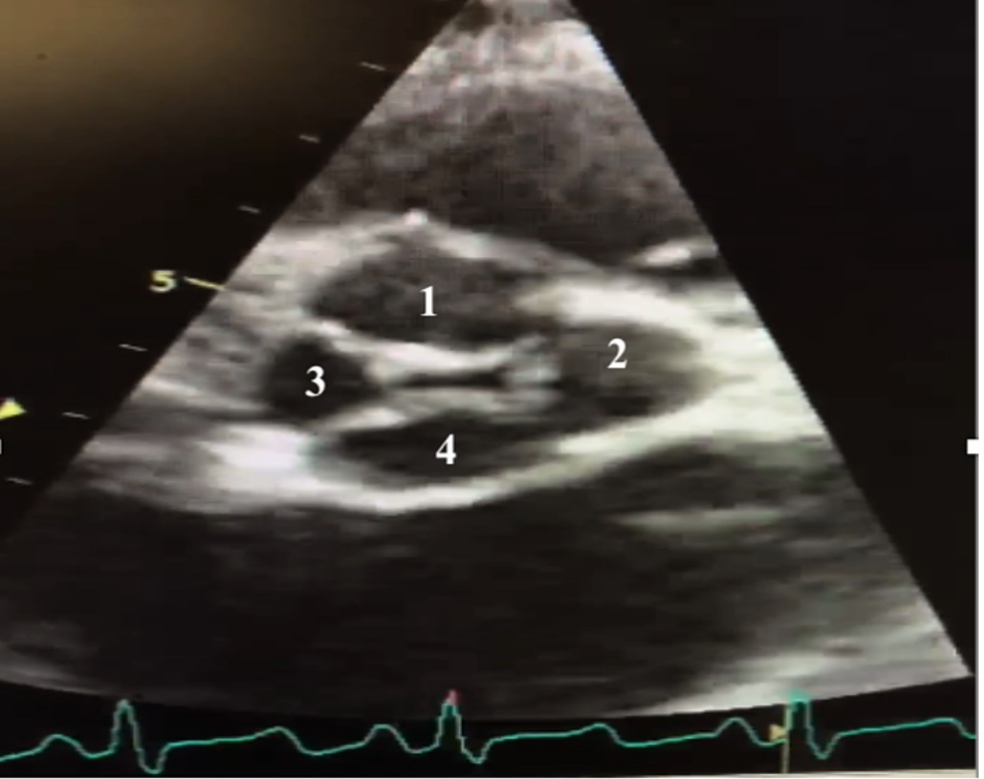
Transesophageal echocardiogram taken two days after admission showing quadricuspid valve (1-4)

An ECG showed sinus tachycardia with left atrial enlargement and left ventricular hypertrophy with repolarization abnormalities. CXR (Figure 4) was significant for cardiomegaly and pulmonary edema. A computed tomography angiography (CTA) of the chest ruled out pulmonary embolism and revealed cardiomegaly, mild right pulmonary effusion, diffuse interstitial edema, and chronic pulmonary obstructive disease with diffuse emphysematous changes. A TEE (Figure 5) done on day 2 of hospitalization showed a severely dilated left ventricular cavity with severely reduced left ventricular systolic function. EF was 25-30%. The aortic valve was quadricuspid and showed moderate to severe regurgitation. There was global chamber dilation and the right ventricular systolic function was mildly reduced. A nuclear stress test (Lexi Scan) was done to rule out ischemic cardiomyopathy, which showed a negative myocardial perfusion study for evidence of ischemia and infarction. Left and right heart catheterization with coronary angiography showed normal coronaries, global hypokinesis, and 3+ aortic regurgitation.

The patient was diuresed and placed on strict input/output, daily weights, and a low sodium diet. After she was clinically stable, she was started on GDMT. Cardiothoracic surgery was consulted, and an aortic valve replacement was recommended, but the patient had poor dentition and needed to have dental extraction prior to the valve replacement. She eventually had the valve replacement done as an outpatient. For the emphysematous changes found on the CTA chest, alpha 1 antitrypsin was within normal limits (=173), and urine legionella antigen testing and urine Streptococcus pneumoniae were negative. Sputum culture and acid-fast bacilli respiratory culture were also negative. She was referred to outpatient pulmonology.

## Discussion

Normal aortic valves have three cusps, but cases of unicuspid, bicuspid, and quadricuspid valves have been reported. The first QAV was identified during an autopsy by Balington in 1862 [3]. A study by Tsang et al. in 2015 performed on 357,228 patients at Mayo Clinic found that 52% of patients with QAV were females (male-to-female ratio, 1:1.08) [4]. The mechanism of development of QAV remains unknown, but many theories have been proposed, including septation of the conotruncus, septation of a normal valve cushion due to an inflammatory process, or division of one of the mesenchymal ridges forming the three aortic cusps [5].

**Figure 6:**
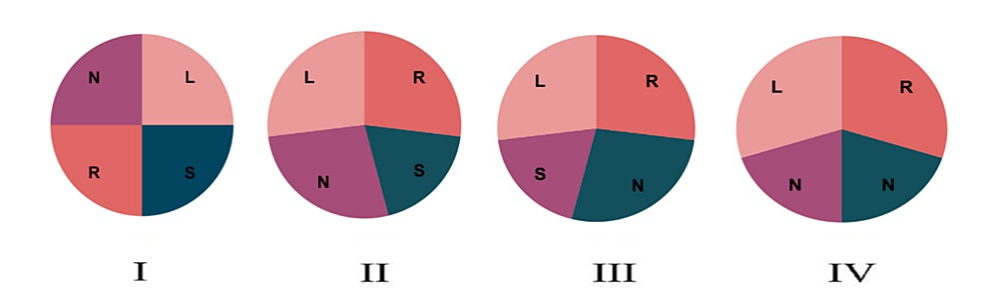
Nakamura et al. classification of the quadricuspid aortic valve Type I: The supernumerary cusp is between the right and left coronary cusps. Type II: The supernumerary cusp is between the right and noncoronary cusps. Type III: The supernumerary cusp is between the left and noncoronary cusps. Type IV: The supernumerary cusp cannot be identified as there are two small equal sized cusps [7]. L: left coronary cusp; N: noncoronary cusp; R: right coronary cusp; S: supernumerary cusp.

Hurwitz and Roberts [6] classified the QAV into seven subtypes. (i) type A: QAV with four equal-sized cusps; (ii) type B: QAV with three equal-sized cusps and one smaller cusp; (iii) type C: QAV with two equal larger cusps and two equal smaller cusps; (iv) type D: QAV with one larger cusp, two intermediate-sized cusps, and one smaller cusp; (v) type E: QAV with three equal-sized cusps and one larger cusp; (vi) type F: QAV with two equal-sized cusps and two unequal smaller cusps; and (vii) type G - QAV with four unequal cusps. Nakamura et al. [7] created a simplified classification of QAV. Here, QAV was classified into four subtypes. These subtypes are: (i) type I: the supernumerary cusp is between the right and left coronary cusps; (ii) type II: the supernumerary cusp is between the right and noncoronary cusps; (iii) type III: the supernumerary cusp is between the left and noncoronary cusps; (iv) type IV: the supernumerary cusp cannot be identified as there are two small equal-sized cusps. When studying 42 patients with QAV, Nakamura found the four subtypes of QAV at 23%, 30.9%, 7.2%, and 4.9%, respectively (Figure 6).

The most common congenital cardiac anomaly is the bicuspid aortic valve (BAV) [8]. It is found in 1-2% of the general population and is known to cause early aortic calcification, leading to early aortic stenosis [8]. In contrast to BAV, aortic insufficiency has been reported as the most common aortic valve abnormality in QAV [2]. In a retrospective study done on patients with QAV, the following abnormalities and frequencies were noted: regurgitant in 74.7%, combined stenosis and regurgitation in 8.4%, stenosis alone in 0.7%, and a normally functioning valve in 16.2% [1]. The exact mechanisms for the development of aortic regurgitation in patients with QAV are not completely understood, but the anatomical abnormalities of the cusps can cause unequal shear stress, which results in leaflet fibrosis and incomplete coaptation, leading to valve insufficiency [3]. An association between the morphological characteristics of QAV and the severity of aortic regurgitation has been established [1]. Mild aortic regurgitation was seen in QAV with four equal-sized leaflets as they were less likely to develop fibrous thickening [9]. However, QAV with a smaller cusp was found to develop severe aortic insufficiency. This is thought to be due to the development of unequal stress distribution leading to fibrosis and abnormal coaptation [9].

While QAV is normally an isolated anomaly, 18-32% [1] of the patients present with associated congenital cardiac defects. The most common associated abnormalities are coronary artery and coronary ostium anomalies [1]. Other associated cardiac defects include: ventral septal defect, hypertrophic nonobstructive cardiomyopathy, patent ductus arteriosus, pulmonary valve stenosis, mitral valve malformation, and subaortic fibromuscular stenosis [9].

Despite QAV being congenital, patients can present with symptoms later in their life [3]. The clinical presentation depends on the functionality of the QAV and the presence of associated disorders [1]. Patients present with palpitations, chest pain, shortness of breath, fatigue, pedal edema, syncopy, heart failure, and in some cases, sudden cardiac death [1]. Previously, QAV was seen only during surgeries and autopsies, but in 1984, two-dimensional TTE was used to diagnose the condition [1]. Today echocardiogram is the preferred diagnostic tool for QAV [9]. TTE aids in diagnosing QAV and assessing the severity of the aortic insufficiency, left ventricular size and function, and the presence of associated cardiac anomalies. However, TEE is more sensitive in capturing all the details missed by TTE and even allows clear visualization of the coronary ostia [9]. Furthermore, computed tomography (CT scan) and cardiac magnetic resonance imaging (MRI) have also been used to diagnose QAV [3].

Asymptomatic patients with incidental QAV findings must be followed regularly as progression to aortic regurgitation is highly likely [5]. Surgical repair of the aortic valve is the gold standard treatment [3]. Indications for surgical repair include severe aortic insufficiency, severe aortic stenosis, or a malfunctioning QAV accompanied by other lesions like left coronary ostium occlusion [1]. Aortic valve repair is preferred over valve replacement as patients present relatively young and valve replacement puts them at high risk of thromboembolism, prosthetic valve degeneration, infective endocarditis, and bleeding [1]. Tricuspidalization is the most common technique to repair the aortic valve [1]. A study in 2014 by Song et al. concluded that tricuspidalization showed satisfactory early and mid-term results [10].

## Conclusions

QAV is a rare congenital cardiac anomaly seen in less than 0.01% of autopsies. Despite being a congenital condition, patients remain asymptomatic until their fourth to fifth decade of life. The development of severe aortic regurgitation is highly likely and, hence, patients diagnosed incidentally must be followed regularly even if asymptomatic. Surgical aortic repair is the standard treatment in cases of severe aortic regurgitation.
